# Supplementary figures and images for: Protection of cultured brain endothelial cells from cytokine-induced damage by α-melanocyte stimulating hormone
Source: PeerJ. 2018 May 15;6:e4774. doi: 10.7717/peerj.4774 (PMC5958884; doi:10.7717/peerj.4774)

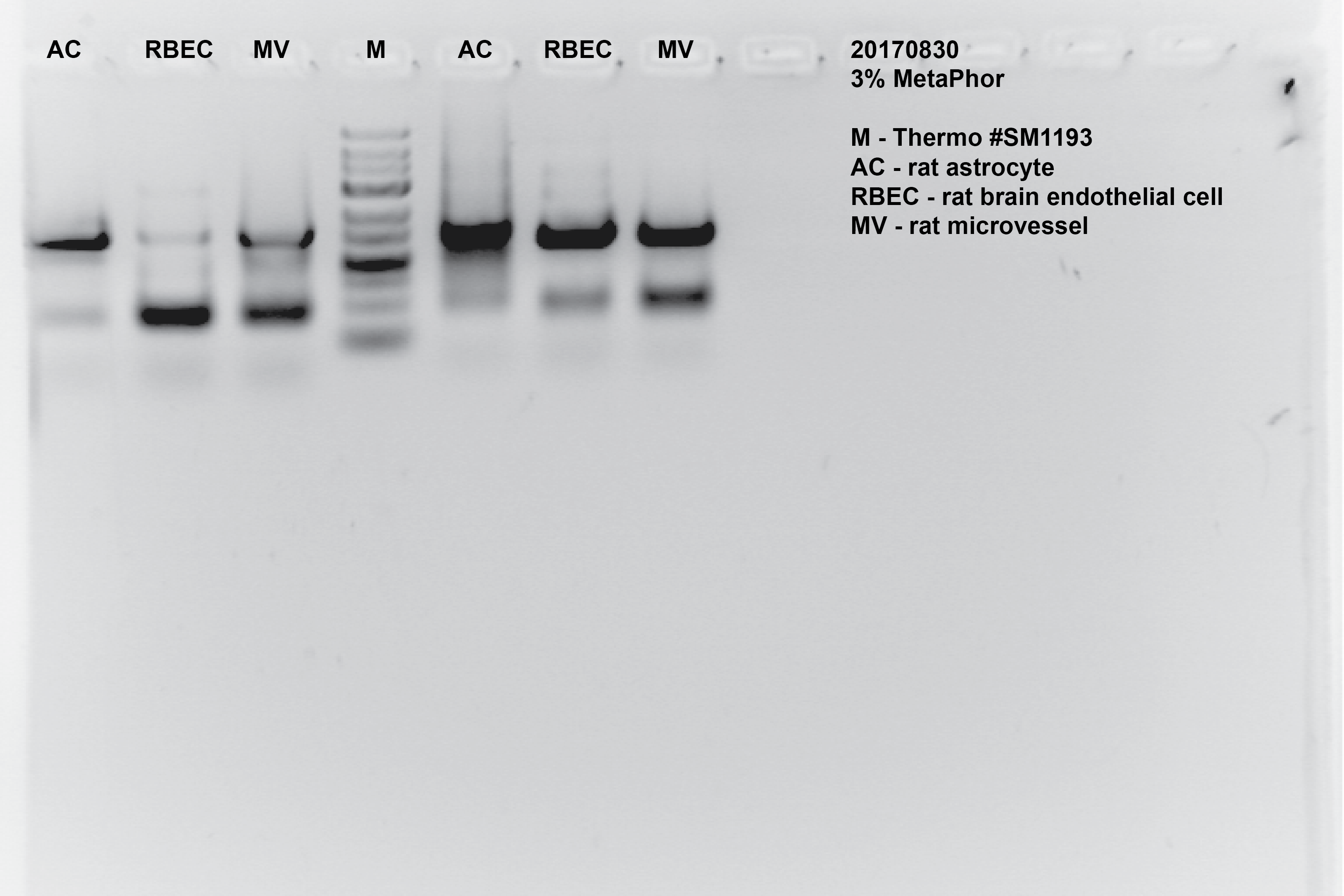

Supplement: Supplemental Information 2 — PCR products for MC1R and beta-actin on a 3% MetaPhore gel. [file peerj-06-4774-s002.png]
